# Specificity of the *STAT4* Genetic Association for Severe Disease Manifestations of Systemic Lupus Erythematosus

**DOI:** 10.1371/journal.pgen.1000084

**Published:** 2008-05-30

**Authors:** Kimberly E. Taylor, Elaine F. Remmers, Annette T. Lee, Ward A. Ortmann, Robert M. Plenge, Chao Tian, Sharon A. Chung, Joanne Nititham, Geoffrey Hom, Amy H. Kao, F. Yesim Demirci, M. Ilyas Kamboh, Michelle Petri, Susan Manzi, Daniel L. Kastner, Michael F. Seldin, Peter K. Gregersen, Timothy W. Behrens, Lindsey A. Criswell

**Affiliations:** 1 Rosalind Russell Medical Research Center for Arthritis, University of California San Francisco, San Francisco, California, United States of America; 2 National Institute of Arthritis and Musculoskeletal and Skin Diseases, Bethesda, Maryland, United States of America; 3 Feinstein Institute for Medical Research, North Shore L.I.J. Health System, Manhasset, New York, United States of America; 4 Genentech, Inc., South San Francisco, California, United States of America; 5 Broad Institute of Harvard and the Massachusetts Institute of Technology, Cambridge, Massachusetts, United States of America; 6 Division of Rheumatology, Immunology and Allergy, Brigham and Women’s Hospital, Harvard Medical School, Cambridge, Massachusetts, United States of America; 7 University of California Davis, Davis, California, United States of America; 8 University of Pittsburgh, Pittsburgh, Pennsylvania, United States of America; 9 Johns Hopkins University School of Medicine, Baltimore, Maryland, United States of America; University of Michigan, United States of America

## Abstract

Systemic lupus erythematosus (SLE) is a genetically complex disease with heterogeneous clinical manifestations. A polymorphism in the *STAT4* gene has recently been established as a risk factor for SLE, but the relationship with specific SLE subphenotypes has not been studied. We studied 137 SNPs in the *STAT4* region genotyped in 4 independent SLE case series (total n = 1398) and 2560 healthy controls, along with clinical data for the cases. Using conditional testing, we confirmed the most significant *STAT4* haplotype for SLE risk. We then studied a SNP marking this haplotype for association with specific SLE subphenotypes, including autoantibody production, nephritis, arthritis, mucocutaneous manifestations, and age at diagnosis. To prevent possible type-I errors from population stratification, we reanalyzed the data using a subset of subjects determined to be most homogeneous based on principal components analysis of genome-wide data. We confirmed that four SNPs in very high LD (r^2^ = 0.94 to 0.99) were most strongly associated with SLE, and there was no compelling evidence for additional SLE risk loci in the *STAT4* region. SNP rs7574865 marking this haplotype had a minor allele frequency (MAF) = 31.1% in SLE cases compared with 22.5% in controls (OR = 1.56, p = 10^−16^). This SNP was more strongly associated with SLE characterized by double-stranded DNA autoantibodies (MAF = 35.1%, OR = 1.86, p<10^−19^), nephritis (MAF = 34.3%, OR = 1.80, p<10^−11^), and age at diagnosis<30 years (MAF = 33.8%, OR = 1.77, p<10^−13^). An association with severe nephritis was even more striking (MAF = 39.2%, OR = 2.35, p<10^−4^ in the homogeneous subset of subjects). In contrast, *STAT4* was less strongly associated with oral ulcers, a manifestation associated with milder disease. We conclude that this common polymorphism of *STAT4* contributes to the phenotypic heterogeneity of SLE, predisposing specifically to more severe disease.

## Author Summary

Systemic lupus erythematosus is a chronic disabling autoimmune disease, most commonly striking women in their thirties or forties. It can cause a wide variety of clinical manifestations, including kidney disease, arthritis, and skin disorders. Prognosis varies greatly depending on these clinical features, with kidney disease and related characteristics leading to greater morbidity and mortality. It is also complex genetically; while lupus runs in families, genes increase one’s risk for lupus but do not fully determine the outcome. It is thought that the interactions of multiple genes and/or interactions between genes and environmental factors may cause lupus, but the causes and disease pathways of this very heterogeneous disease are not well understood. By examining relationships between subtypes of lupus and specific genes, we hope to better understand how lupus is triggered and by what biological pathways it progresses. We show in this work that the *STAT4* gene, very recently identified as a lupus risk gene, predisposes specifically to severe manifestations of lupus, including kidney disease.

## Introduction

Systemic lupus erythematosus (SLE) (OMIM 152700) is a disabling and chronic autoimmune disease with remarkable heterogeneity. The eleven classification criteria for SLE established by the American College of Rheumatology [1] – any four of which can confirm classification as SLE – include arthritis, renal disease, mucocutaneous manifestations, photosensitivity, neurological disorders, production of a variety of autoantibodies, and hematological disorders. Many of these characteristics are correlated, and may indicate different underlying disease mechanisms. SLE also has an established but complex genetic component [2]. Understanding the relationships between SLE risk genes and subtypes of the disease may help to elucidate disease mechanisms and pathways.

Recently, a polymorphism of the *STAT4* gene on chromosome 2q has been strongly implicated in the risk for both SLE and rheumatoid arthritis [3]. We investigated whether variation in *STAT4* contributes to the heterogeneity of SLE. Using 4 independent SLE case series, a large set of healthy controls, and two independent sets of genotypes for the *STAT4* region on these subjects, we have found strong evidence that this is the case. In particular, we have found that the *STAT4* susceptibility polymorphism is associated with more severe disease manifestations, including nephritis and early disease onset. It is also strongly associated with SLE characterized by double-stranded DNA autoantibody production.

## Methods

### Subjects

SLE cases were obtained from four sources. Patients from the University of California, San Francisco (UCSF) were participants in the UCSF Lupus Genetics Project and were recruited from UCSF Arthritis Clinics and private rheumatology practices in northern California, as well as by nationwide outreach [4]. SLE patients contributed by the Autoimmune Biomarkers Collaborative Network (ABCoN) [5] were recruited from the Hopkins Lupus cohort [6]. A third case series was part of the Multiple Autoimmune Disease Genetics Consortium (MADGC) collection [7]. Finally, a fourth set of cases recruited from the Pittsburgh Lupus Registry were obtained from the University of Pittsburgh [8]. Only subjects of self-described European descent were retained. Unrelated controls of European ancestry were from the New York Health Project (NYHP) [9] (http://www.amdec.org/amdec_initiatives/nycp.html). The study populations are a superset of those recently used to establish a link between SLE and *STAT4* [3], with the addition of the University of Pittsburgh cases and more than doubling the number of NYHP controls (see Table 1). The Institutional Review Boards of all investigative institutions approved these studies, and all cases and controls gave written informed consent.

**Table 1:** Summary of available genotype and phenotype data* by cohort and genotyping platform.

	Illumina 550K genotyped	Sequenom genotyped	Genotyped on both platforms	Genotyped on either platform	Phenotype data available
UCSF** cases	611	583	580	614	614
ABCoN** cases	330	347	330	347	345
MADGC** cases	116	105	103	118	118
U. Pittsburgh cases	319	0	0	319	319
**Total cases**	**1376**	**1035**	**1013**	**1398**	**1396**
**NYHP** ** ** controls**	**1762**	**1243**	**445**	**2560**	**N/A**

After removal of duplicate samples and first-degree relatives, but prior to other quality control filters.

UCSF = University of California, San Francisco; ABCoN = Autoimmune Biomarkers Collaborative Network; MADGC = Multiple Autoimmune Disease Genetics Consortium; NYHP = New York Health Project.

Clinical data for the cases was obtained from medical records which were reviewed and tabulated at each institution. We chose to examine the ACR criteria [1] (http://www.rheumatology.org/publications/classification/SLE/sle.asp) and age at diagnosis, categorizing the age at diagnosis for association analyses. The mean and median for age at diagnosis were 34 and 32, respectively; we chose a cutoff for early diagnosis of under 30 years of age versus greater than or equal to 30 years of age. We also chose to examine production of autoantibodies to double-stranded DNA (anti-dsDNA), as this is typically associated with severe disease and this was available in the clinical data from all sites. Finally, we used more detailed nephritis information available for the UCSF and ABCoN cohorts, namely a characterization of those patients with severe nephritis as defined by end-stage renal disease or histopathologic evidence of severe, progressive renal disease on renal biopsy.

### Genotyping and SNP Selection

Genotype data were obtained from two parent studies (see Table 1). The four SLE case series and 1762 NYHP controls were genotyped using the Illumina HumanHap550 array as part of a genome-wide association study of SLE [10]. In addition, three of the case series (UCSF, ABCoN, and MADGC) and 1243 NYHP controls were genotyped for 67 SNPS in the STAT1/STAT4 region of chromosome 2q as part of a case-control study of *STAT4* and two systemic autoimmune diseases, rheumatoid arthritis and SLE [3]. Selection and genotyping of these 67 fine-mapping SNPs was done by the National Institute for Arthritis and Musculoskeletal and Skin Diseases (NIAMS), using Sequenom MassARRAY Technology as previously described [3]. From the Illumina 550K panel, 91 contiguous SNPs from the *STAT1/STAT4* region, extended with flanking regions 200kb on either side, were selected; of these, 45 were contained in the same region as the 67 SNPs, with 21 of those being identical. Coverage of these SNPs was analyzed using Tagger [11] in Haploview [12] with an r^2^ threshold of 0.8 and pairwise tagging, based on the HapMap Phase II data from CEU (CEPH residents of Utah with ancestry from northern and western Europe) with minor allele frequency>0.05.

Duplicate genotyping enabled an analysis of SNP concordance between the two genotyping methods, and inclusion of genotypes that were called by either method. Conflicting genotype calls were dropped from analyses when using combined data.

### Statistical Analysis

Subjects were first removed for whom there was evidence of duplication or relatedness in the Illumina 550K data, using IBS estimation in PLINK [13] (http://pngu.mgh.harvard.edu/purcell/plink). For the choice of which sample to remove, preference was first based on availability of phenotype data, and then on overall genotyping call rate. SNPs were removed from analysis that had a minor allele frequency less than 5%, greater than 10% missing genotypes, or Hardy-Weinberg equilibrium p<0.001 in controls.

In order to choose loci to examine for phenotype analyses, we performed allelic and conditional tests. These analyses were performed separately for the 91 SNPs from the Illumina 550K panel and the 67 Sequenom SNPs, since they contained overlapping but different sets of SNPs (Table S1) and subjects (Table 1), and large amounts of missing data could bias haplotype estimation. We also analyzed the full set of 137 SNPs together using only the subset of subjects who were genotyped on both platforms. For each analysis, subjects were removed that had less than 90% genotyping. We first conducted allelic tests of cases and controls using PLINK [13] and selected those that had p<0.005; at this first screening stage we used a liberal p-value, considering that there are well over 10 independent haplotype blocks in the complete region. To eliminate redundant SNPs having effects only due to linkage disequilibrium, we then performed conditional analysis using WHAP [14] (http://pngu.mgh.harvard.edu/purcell/whap).

SNP rs7574865 was chosen for phenotype analysis based on the allelic and conditional analyses (see Results). SNP rs7574865 was genotyped on both platforms with a very high rate of concordance (see Results), so genotypes from both platforms were combined for the phenotype analysis of rs7574865; the single subject for whom the calls conflicted was dropped. We first performed case-only analyses (e.g. presence of renal disorder versus no renal disorder) to establish which subphenotypes are associated with rs7574865 variation. We then performed case-control analyses (e.g. SLE with renal disorder versus controls) to examine the risk that is conferred by rs7574865 on subtypes of SLE characterized by each of those subphenotypes. In both sets of analyses, first bivariate odds ratios (ORs) and 95% confidence limits were determined for each subphenotype. To correct for variability among strata when combining data from different cohorts, we used Mantel-Haenszel tests and combined ORs. In order to investigate the possibility of associations with unknown but common underlying disease mechanisms, principal components analysis (PCA) was performed using all subphenotypes except severe nephritis (a subclass of nephritis, and available only for the UCSF and ABCoN case series). Values for the first two principal components (PCs) were evaluated as above as additional subphenotypes, categorized by positive or negative.

To address the concern that case-control studies may give spurious associations due to undetected population admixture or population substructure differences between cases and the controls, we utilized ancestry data for the Illumina 550K genotyped subjects. Ancestry was derived from ancestry-informative markers (AIMs) contained in the Illumina 550K panel. First a set of 235 AIMs was used to estimate percent European ancestry, using STRUCTURE [15]. For those subjects with >90% European ancestry, another set of 1409 EUROSTRUCTURE [16] AIMs was used to estimate percent Northern European versus Southern ancestry. Finally, a subset of 1253 subjects (751 cases and 502 controls) was identified that was homogeneous based on the first four PCs determined by PCA using the 550K panel and EIGENSTRAT[17] software. Minimum covariance determinant (MCD) estimators of PC location and scatter were calculated using R [18]; outliers were then determined using robust Mahalanobis distance. The procedure was applied in two steps, first using both cases and controls (significance level α = 0.05), and then using the case-only robust estimators of location and scatter (α = 0.10), which led to a more homogeneous case-control sample set. The λ_gc_ was decreased from 1.256 to 1.045 for the homogeneous set when assessed using the 550K panel (see Figure S1).

We analyzed the associations between ≥90% European versus <90% European and ≥90% Northern European versus <90% Northern European ancestry and rs7574865 in controls, using an allele-based exact test. We also reanalyzed all tests using the homogeneous subset of subjects. Finally, in multivariate analysis we adjusted for ancestry, sex, and disease duration. For this multivariate analysis, ancestry was a 3-category variable as follows: 1) <90% European, 2) ≥90% European and ≥90% Northern European, and 3) ≥90% European and <90% Northern European. We chose this coding due to the highly skewed distribution of continuous ancestry, and the collinearity between the European and Northern European variables.

Since we are examining associations for 13 phenotypes, the issue of multiple testing must be considered. However, since these are not independent phenotypes, a simple Bonferroni correction of α = 0.05/13 = 0.004 is clearly overly conservative, while an unadjusted α = 0.05 is clearly liberal. For this reason we have chosen to present unadjusted p-values so that these may be directly interpreted by the reader.

Stata 9.2 (http://www.stata.com/) was used for correlations, odds ratios and p-values, Mantel-Haenszel tests and combined ORs, phenotype principal components analysis, and multivariate logistic regressions.

## Results

### Subjects and Phenotypes

The numbers of independent cases and controls in each cohort and a summary of available genotype and phenotype data are listed in Table 1. For overlapping SNPs, including rs7574865, there were 1396 genotyped cases with phenotype data, and 2560 genotyped healthy controls. A summary of subphenotypes by cohort is presented in Table 2. There were significant differences among the cohorts for all phenotypes except neurologic disorder and age at diagnosis less than 30 years old.

**Table 2:** SLE phenotype status by cohort.

Phenotypes*	UCSF**	ABCON**	MADGC**	U. PITTSBURGH	p-value***
Anti-nuclear autoantibodies	572/598 (95.7%)	328/343 (95.6%)	109/118 (92.4%)	314/319 (98.4%)	0.018
Arthritis	460/614 (74.9%)	251/345 (72.8%)	101/118 (85.6%)	289/319 (90.6%)	2.9E-10
Immunologic disorder	359/614 (58.5%)	272/345 (78.8%)	83/118 (70.3%)	236/319 (74.0%)	7.3E-11
Photosensitivity	484/614 (78.8%)	224/345 (64.9%)	94/118 (79.7%)	187/317 (59.0%)	5.5E-11
Hematologic disorder	387/614 (63.0%)	238/345 (69.0%)	65/118 (55.1%)	164/317 (51.7%)	3.2E-05
Anti-dsDNA autoantibodies†	284/587 (48.4%)	184/344 (53.5%)	59/118 (50.0%)	136/319 (42.6%)	0.047
Malar rash	282/614 (45.9%)	197/345 (57.1%)	67/118 (56.8%)	133/283 (47.0%)	0.0026
Oral ulcers	189/614 (30.8%)	207/345 (60.0%)	47/118 (39.8%)	173/318 (54.4%)	1.6E-20
Serositis	185/614 (30.1%)	168/344 (48.8%)	49/118 (41.5%)	140/316 (44.3%)	2.0E-08
Diagnosis<30 years	233/605 (38.5%)	137/344 (39.8%)	36/90 (40.0%)	129/319 (40.4%)	0.94
Renal disorder	143/614 (23.3%)	116/345 (33.6%)	37/118 (31.4%)	96/318 (30.2%)	0.0033
Severe nephritis‡	71/614 (11.6%)	38/345 (11.0%)	NA	NA	9.6E-05
Discoid rash	39/614 (6.4%)	56/345 (16.2%)	18/118 (15.3%)	15/283 (5.3%)	1.6E-07
Neurologic disorder	60/614 (9.8%)	30/345 (8.7%)	10/118 (8.5%)	29/317 (9.2%)	0.95

See http://www.rheumatology.org/publications/classification/SLE/sle.asp for phenotype definitions.

See Table 1 cohort definitions.

Global exact test for association between phenotype status and cohort membership.

**†:** Historical presence of positive anti-dsDNA test.

**‡:** Presence of end-stage renal disease or histopathologic evidence of severe, progressive renal disease on renal biopsy.

Some of these phenotypes are highly correlated; in particular anti-dsDNA is a subcriterion for the ACR immunologic criterion, and is associated with renal disease. Pairwise correlation coefficients, for those pairs having ρ>0.1, are shown in Table 3. All p-values for these pairs were ≤0.0001. In principal components (PC) analysis of the phenotype data, the top 3 components of the first PC are anti-dsDNA, the immunologic criterion, and renal disease. The top 3 components of the second PC are malar rash, photosensitivity, and discoid rash. Variables corresponding to the first and second PCs were included in phenotype analyses (see Methods).

**Table 3:** Correlation coefficient rho for phenotype pairs with rho>0.1 and first two principal components.

Phenotypes*	1st PC**	2nd PC**	Renal disorder	Immunologic disorder	Anti-dsDNA autoantibodies	Hematologic disorder	Serositis	Malar Rash	Photo-sensitivity
Renal disorder	0.59	0.21							
Immunologic disorder	0.76	−0.24	0.22						
Anti-dsDNA autoantibodies	0.80	−0.19	0.28	0.65					
Hematologic disorder	0.36	-	0.15	0.10	0.14				
Serositis	0.20	0.34	-	-	-	-			
Malar rash	0.14	0.66	0.10	-	-	-	-		
Photosensitivity	−0.22	0.48	-	−0.15	−0.11	-	-	0.19	
Oral ulcers	−0.10	0.34	-	-	-	-	0.11	0.11	-
Age at diagnosis	−0.50	−0.39	−0.28	−0.17	−0.20	−0.13	−0.11	−0.19	-
Discoid rash	-	0.42	-	-	-	-	-	-	0.12
Anti-nuclear autoantibodies	0.17	−0.12	-	-	0.13	-	-	-	-
Neurologic disorder	0.20	0.21	-	-	-	-	-	-	-

Significance is p<0.0001 for all pairs shown. Blank cells are given in the lower triangular matrix.

See http://www.rheumatology.org/publications/classification/SLE/sle.asp for phenotype definitions.

PC = principal component.

### SNP Coverage, Concordance, and Merging

**Figure 1:**
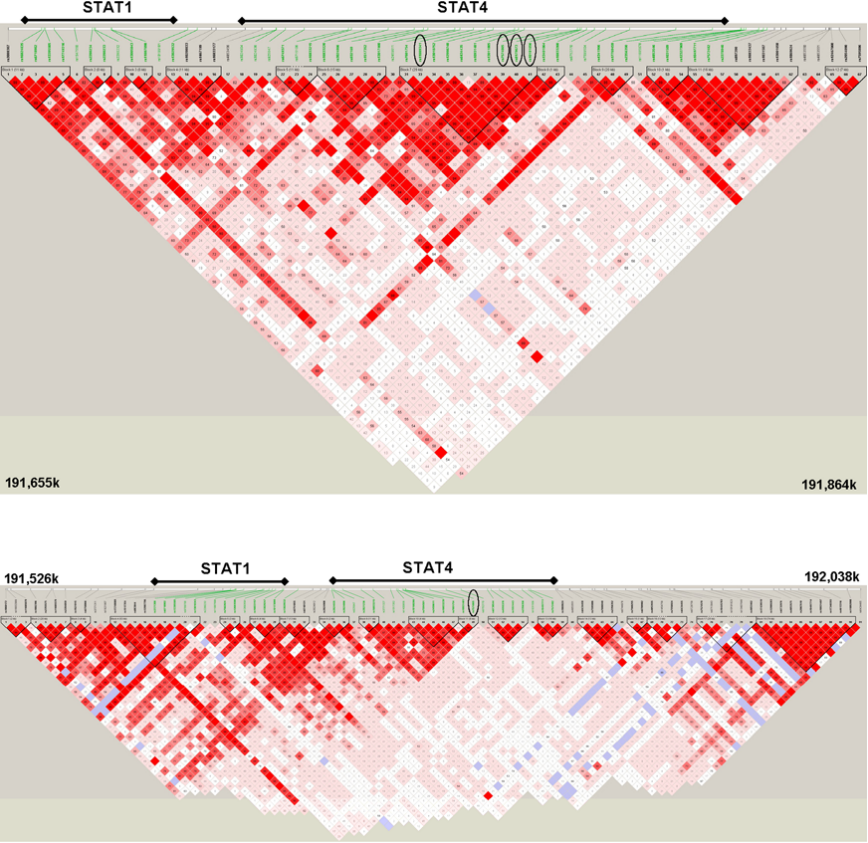
Haploview linkage disequilibrium map of D’ for 67 Sequenom STAT1/STAT4 SNPs in 2278 study subjects. (A) Green markers are in STAT1 and STAT4 genes as indicated. Four SNPs comprising the top SLE risk haplotype, from allelic and conditional analyses (Table 4 (C)), are circled. (B) Haploview linkage disequilibrium map of D’ for 91 Illumina 550K STAT1/STAT4 extended region SNPs in 3138 study subjects. Green markers are in STAT1 and STAT4 genes as indicated. The top SLE risk SNP rs7574865 from allelic and conditional analyses (Table 4 (A)) is circled.

Of the 67 Sequenom SNPs (shown with the study genotypes in Figure 1A), 62 passed quality control filters, including MAF≥5%. These 62 SNPs had 86% coverage of the common variation (MAF≥5%) in the *STAT1/STAT4* region. In the Illumina 550K panel (shown with the study genotypes in Figure 1B), 77 out of 91 SNPs passed quality control and had 83% coverage of the extended region obtained by adding flanking markers 200kb on either side of the Sequenom *STAT1-STAT4* region. A complete list of SNPs on both platforms passing our quality control criteria, along with their MAF and percentage genotyped, is provided in Table S1.

**Table 4:** Allelic and conditional tests for all SNPs with p<0.005.

	SNP	Single-marker Allelic P-value	Single-marker Allelic OR	P Conditioned on top SNP(s) in bold
A) Illumina 550K n = 3132	rs3821236	3.1E-08	1.40	0.73
	rs16833215	0.00042	1.21	0.45
	rs1517352	0.00028	1.21	0.025
	rs10168266	1.4E-08	1.41	0.81
	rs7601754	0.00015	0.77	0.062
	rs10931481	1.9E-07	1.32	0.050
	**rs7574865**	**8.2E-14**	**1.54**	NA
	rs6752770	0.0029	1.18	0.60
	rs2356350	0.0052	1.16	0.55
B) Illumina 550K, Homogeneous subset n = 1252	rs3821236	0.0011	1.40	0.71
	rs10168266	0.0034	1.34	0.38
	rs7601754	0.0016	0.71	0.039
	rs10931481	0.0021	1.31	0.058
	**rs7574865**	**2.9E-06**	**1.57**	NA
C) Sequenom STAT4 fine map n = 2083	rs1547550	0.0032	1.21	0.10
	rs16833177	0.0032	1.25	0.88
	rs7601754	0.00054	0.75	0.075
	**rs11889341**	**1.3E-08**	**1.49**	NA
	rs12998748	0.0021	0.71	0.084
	rs6434435	0.0034	0.78	0.22
	rs10931481	9.2E-06	1.34	0.56
	rs13011805	0.0024	0.72	0.092
	**rs7574865**	**1.1E-08**	**1.50**	NA
	**rs8179673**	**2.2E-08**	**1.48**	NA
	**rs10181656**	**1.1E-08**	**1.50**	NA
	rs16833260	4.7E-05	1.30	0.29
	rs6752770	0.0015	1.24	0.37
D) Illumina 550K and Sequenom combined for subjects having both typing n = 1351	rs10168266	0.0032	1.36	0.50
	**rs11889341**	**0.00019**	**1.44**	NA
	rs10931481	0.0010	1.34	0.75
	**rs7574865**	**0.00021**	**1.44**	NA
	**rs8179673**	**6.9E-05**	**1.47**	NA
	**rs10181656**	**5.1E-05**	**1.48**	NA
	rs16833260	0.0033	1.30	0.51

Allelic odds ratios (ORs) and p-values from PLINK, p-values conditional on bold SNPs from Whap [14]. Subjects with <90% genotyping are excluded for each analysis. For (C) and (D), the four top SNPs in bold are indistinguishable, i.e. any one fully determines the others in >99% of haplotypes.

We examined the concordance between calls for the 21 overlapping SNPs and 1458 subjects who were genotyped using both methods. Results for this are shown in Supplementary Table S2. The average and minimum agreement were 99.90% and 99.65%, respectively. In particular for SNP rs7574865, the agreement was 99.93%. Given this high rate of concordance, we chose to merge genotype data for rs7574865 for the phenotype analyses (see below).

### Allele Tests and Conditional Analysis

Table 4 contains allelic p-values before and after conditioning on the most significant SNP, for those with initial allelic p-values of 0.005 or less. We did four separate conditional analyses: (A) subjects and SNPs genotyped on the Illumina 550K; (B) the genetically homogeneous subset of subjects (see Methods) typed on the Illumina 550K; (C) subjects and SNPs genotyped on the Sequenom platform; and (D) all SNPs for those subjects that were genotyped on both platforms. In the Illumina 550K panel, rs7574865 (circled in Figure 1B) was the most significant SNP in both the full set of subjects and the homogeneous subset (see Methods). In the Sequenom 67-SNP set and in the combined set, the 4 top SNPs were in high LD (D’ = 0.97 to 0.99, r^2^ = 0.94 to 0.99) and made up a 4-marker haplotype for which the components could not be independently analyzed (circled in Figure 1A). Of the estimated individual haplotypes of these 4 markers, over 99% were either CGTC or TTCG, so that any one SNP fully determined the other 3 in the vast majority of subjects.

The conditional p-values of Table 4 test the significance of each SNP conditional on the values of the top SNP(s) which are given in bold. While there were some results of borderline significance, they were neither strong nor consistent across the different analyses. The only compelling evidence after conditioning was for the 4-locus haplotype above. Since any of the 4 SNPs serves as a marker of this haplotype and rs7574865 is contained in both genotyping sets, we chose to carry out phenotype analysis using this marker for maximum power.

### Ancestry Variability of rs7574865

We examined the minor allele frequencies for rs7574865 (Table S3) in controls, for subsets as determined by STRUCTURE analyses (see Methods). There were 130 controls with <90% European ancestry, for whom the minor allele frequency was 26.9%, versus 22.4% in the complementary 1601 controls with ≥90% European ancestry (p = 0.11). (The minor allele frequencies of the HapMap populations are 33%, 28%, 16%, and 21%, for the HCB, JPT, YRI, and CEU populations, respectively.) The minor allele frequencies were very similar, 22.1% and 22.6% respectively, for subjects of either <90% or ≥90% Northern European ancestry; thus we did not observe a Northern-Southern European gradient for rs7574865. Analyses were repeated with the homogeneous subset of cases and controls (n = 1253) as described in Methods.

### Phenotype Case-Only Analysis

We first examined each subphenotype for association with rs7574865 within the SLE cases. In unadjusted results (Table S4), only one phenotype showed borderline evidence for heterogeneity of association among the four SLE case series (p = 0.04 for immunologic disorder); thus we retained combined Mantel-Haenszel odds ratios and p-values. The most significant associations were with anti-dsDNA autoantibodies and the first principal component, OR = 1.44 (95% CI 1.23–1.70), p = 10^−5^, and OR = 1.43 (95% CI 1.21–1.70), p = 3×10^−5^, respectively. Severe nephritis, available on a smaller subset of cases, had the highest OR = 1.50 (95% CI 1.11–2.01), p = 0.0075. There was also support for associations with immunologic criteria (OR = 1.24, p = 0.017), renal disorder (OR = 1.23, p = 0.024), age at diagnosis under 30 (OR = 1.22, p = 0.018), and an inverse association with oral ulcers (OR = 0.80, p = 0.0087).

Table 5 contains case-only analyses, for phenotypes having unadjusted p<0.05, repeated first on the homogeneous subset of subjects (see Methods), and also using multivariate adjustment for ancestry, sex, and disease duration. There is consistency in odds ratios throughout and these analyses continue to support the aforementioned phenotypic associations with rs7574865. Some associations are even stronger in the homogeneous subset analysis, for example severe nephritis (OR = 1.79 [95% CI = 1.20–2.67], p = 0.0039), renal disease (OR = 1.48 [95% CI = 1.16–1.88], p = 0.0016), and oral ulcers (OR = 0.62 [95% CI = 0.49–0.79], p = 0.0001).

**Table 5:** rs7574865 association with phenotype status of cases in homogeneous subset (n = 751) and multivariate analyses.

Phenotypes§	Homogeneous OR*	Homogeneous p-value*	Multivariate OR**	Multivariate p-value**
Severe nephritis‡	1.79 (1.20–2.67)	0.0039	1.43 (1.05–1.94)	0.022
Renal disorder	1.48 (1.16–1.88)	0.0016	1.18 (0.98–1.42)	0.074
First PC†>0	1.42 (1.12–1.79)	0.0033	1.37 (1.15–1.63)	0.00047
Anti-dsDNA autoantibodies	1.40 (1.12–1.76)	0.0037	1.41 (1.19–1.67)	7.20E-05
Diagnosis<30 years	1.35 (1.07–1.70)	0.012	1.22 (1.03–1.44)	0.020
Immunologic disorder	1.19 (0.94–1.52)	0.15	1.20 (1.00–1.44)	0.046
Oral ulcers	0.62 (0.49–0.79)	0.00010	0.81 (0.68–0.95)	0.012

**§:** See Table 1 for phenotype definitions.

Mantel-Haenzel odds ratio (OR) and p-value combined across cohorts.

Multivariate logistic regression adjusting for sex; ancestry as a categorical variable (90% or greater Northern European, 90% or greater European but <90% Northern European, not 90% or greater European); and disease duration for all outcomes except age of diagnosis.

**‡:** UCSF and ABCoN only (see definitions Table 1): homogeneous n = 461, multivariate n = 790.

**†:** First principal component of phenotypes (see Methods).

### Subphenotype-Control Analysis

Table 6 shows our primary results, the risk of SLE characterized by each subphenotype versus healthy controls. This illustrates a spectrum of minor allele frequencies for certain subphenotypes of SLE, with the most extreme being severe nephritis, MAF = 38.1% (OR = 2.12 [95% CI = 1.58–2.83], p = 4×10^−7^), anti-dsDNA autoantibodies, MAF = 35.1% (OR = 1.86 [95% CI = 1.63–2.13], p = 6×10^−20^), and the first principal component, MAF = 35.0% (OR = 1.85 [95% CI = 1.62–2.12], p = 10^−19^). In contrast, controls had a MAF of only 22.5% and cases as a whole had a MAF of 31.1%. SNP rs7574865 is also associated with higher risk for SLE with renal disorder (OR = 1.80, MAF = 34.3%), diagnosis under 30 years old (OR = 1.77, MAF = 33.8%), and immunologic disorder (OR = 1.67, MAF = 32.6%). There is also strong evidence that the *STAT4* risk allele is less frequent in SLE with oral ulcers, MAF = 28.8%, which is generally associated with milder disease.

**Table 6:** rs7574865 MAFs and associations in subphenotype cases vs. controls.

	All Subphenotype Cases and Controls			Homogeneous Subset		
Phenotypes§	MAF	OR (95% CI)	p-value	MAF	OR (95% CI)	p-value
Severe nephritis*	38.1%	2.12 (1.58–2.83)	4.1E-07	39.2%	2.35 (1.54–3.56)	5.1E-05
Anti-dsDNA autoantibodies	35.1%	1.86 (1.63–2.13)	6.3E-20	33.6%	1.85 (1.47–2.32)	7.0E-08
First PC**>0	35.0%	1.85 (1.62–2.12)	1.2E-19	34.0%	1.88 (1.49–2.37)	3.8E-08
Renal disorder	34.3%	1.80 (1.53–2.13)	3.4E-12	36.0%	2.05 (1.58–2.65)	2.8E-08
Diagnosis<30 years	33.8%	1.77 (1.53–2.04)	2.6E-14	34.0%	1.89 (1.48–2.41)	1.8E-07
No oral ulcers	33.0%	1.70 (1.50–1.93)	3.3E-16	33.9%	1.87 (1.51–2.33)	4.4E-09
Immunologic disorder	32.6%	1.67 (1.48–1.90)	2.3E-17	31.3%	1.66 (1.35–2.06)	1.1E-06
**All cases**	**31.1%**	1.56 (1.40–1.73)	1.1E-16	**30.0%**	1.56 (1.29–1.90)	3.0E-06
No renal disorder	29.8%	1.47 (1.30–1.65)	2.10E-10	27.5%	1.39 (1.13–1.71)	0.0016
Diagnosis≥30 years	29.5%	1.44 (1.27–1.64)	1.50E-08	27.7%	1.40 (1.13–1.74)	0.0019
Oral ulcers	28.8%	1.40 (1.21–1.61)	4.70E-06	25.0%	1.22 (0.96–1.55)	0.10
No immunologic disorder	27.9%	1.34 (1.14–1.58)	0.00047	27.5%	1.39 (1.08–1.79)	0.010
No anti-dsDNA autoantibodies	27.3%	1.30 (1.13–1.49)	0.00018	26.7%	1.33 (1.06–1.67)	0.013
**Controls**	**22.5%**	-	-	**21.5%**	-	-

Results are 2×2 odds ratios with two-sided Fisher’s exact p-values.

**§:** See Table 1 for phenotype definitions.

Subset with nephritis detail (UCSF and ABCoN).

First principal component of phenotypes.

This analysis was repeated with the genetically-homogeneous subset, again showing even stronger results for severe nephritis, MAF = 39.2% (OR = 2.35 [95% CI 1.54–3.56]), and stronger inverse results for oral ulcers, MAF = 25.0%, versus MAF = 30.0% for all homogeneous cases and MAF = 21.5% for homogeneous controls.

## Discussion

Genotype-phenotype associations between risk alleles and disease subtypes may give insight into disease etiology and mechanisms. Recent results show that rs7574865, a variant allele of *STAT4,* confers an increased risk for both SLE and rheumatoid arthritis (RA) [3],[19], suggesting the involvement of common pathways of pathogenesis among these two autoimmune diseases. *STAT4*-deficiency is associated with accelerated renal disease and increased mortality [20] in a murine lupus model, but with protective effects for arthritis in knockout mice [21]. Since SLE is an extremely heterogeneous disease, with multiple correlated subphenotypes, we sought to investigate whether or not *STAT4* appears to contribute to this phenotypic heterogeneity in human SLE. We believe that our data provide compelling evidence that *STAT4* is associated with more severe SLE manifestations, particularly with nephritis and with the production of autoantibodies to double-stranded DNA. In contrast, other recently-discovered SLE risk polymorphisms do not appear to be strongly associated with severe disease manifestations [10].

There have been recent successes in the study of genotype-phenotype associations in SLE and other autoimmune diseases. For example, *PDCD1* has been shown to be associated with lupus nephritis and anti-phospholipid antibodies in ethnic subgroups [4], and *PTPN22* is primarily associated with anti-cyclic citrullinated peptide (anti-CCP) [22] and rheumatoid factor (RF) [23] autoantibody positive RA. The *STAT4* gene has been shown to be associated with both anti-CCP positive and negative RA [3]; it has not yet been investigated in the context of SLE subphenotypes.

Replication of genotype-phenotype associations can be challenging [24]; a strength of our study is the inclusion of four independent case series. Other strengths include the availability of two overlapping genotype sets in the *STAT4* region for most of the subjects, including genome-wide data to facilitate ancestry analysis, and of course the availability of detailed phenotype data on all four of the case series.

A limitation of our study is that the subjects are of self-reported European ancestry and primarily female. It could be insightful to look at these associations in other populations, particularly since SLE has higher prevalence among African-Americans and other non-European populations [2]. The *STAT4* gene has recently been shown to be associated with RA in a Korean population [19]; however significant associations with subphenotypes – namely age at onset, radiographic progression, and serologic status – were not found.

Another limitation is the inherent difficulty in obtaining accurate phenotype data. Differences between our 4 SLE cohorts may be true differences in patient characteristics, perhaps as a result of differences in selection, but could also be influenced by different methods of assessment and accuracy of individual records. However, although some of the phenotypes we examined are related to disease activity, and may fluctuate naturally or as a result of treatment, we classified SLE patients according to a history of these specific phenotypes. We are encouraged by the fact that our results were quite homogeneous across the different cohorts. Also, any misclassification would presumably be non-differential with respect to genotypes, thus diluting our results rather than causing type I error.

Finally, it is important in genetic studies to protect against false associations due to undetected population substructure. Indeed there were some subjects in our cohort with sizeable non-European ancestry, in spite of being self-reported European, and those had a higher minor allele frequency for rs7574865. However, reanalysis of a more homogeneous subset of subjects of primarily northern European ancestry was very consistent with our overall results. There is even stronger evidence in this subset for relationships between the *STAT4* rs7574865 SNP and nephritis subphenotypes, and for an inverse relationship with oral ulcers.

Since the subphenotypes having the strongest risk conferred by rs7574865 were highly correlated, we included clinical variables based on principal components (PC) analysis to investigate the possibility of common underlying effects. The first PC, associated with the severe manifestations of anti-dsDNA antibodies, nephritis and immunologic abnormalities, had similar associations as those of its components. Severe nephritis was consistently the most strongly associated subphenotype. The second PC, associated with the milder skin disease manifestations of malar rash, photosensitivity, and discoid rash, was not significantly associated with rs7574865 in any analysis.

In summary, our study has identified multiple correlated subphenotypes that are strongly associated with the *STAT4* gene, including nephritis, autoantibodies to double-stranded DNA, and early age at diagnosis. The next challenge is identifying how these correlated features fit into causal pathways, and therefore to help elucidate the complex etiology of SLE.

## Supporting Information

Figure S1 Robust distance method reduces heterogeneity of subject sets.
(0.50 MB TIF)

Table S1 MAFs and percent genotyping for all SNPs.
(0.27 MB DOC)

Table S2 SNP Concordance.
(0.05 MB DOC)

Table S3 SNP rs7574865 by ancestry.
(0.03 MB DOC)

Table S4 rs7574865 case-only associations.
(0.04 MB DOC)

## References

[1] Tan EM, Cohen AS, Fries JF, Masi AT, McShane DJ (1982). The 1982 revised criteria for the classification of systemic lupus erythematosus.. Arthritis Rheum.

[2] Tsao BP (2003). The genetics of human systemic lupus erythematosus.. Trends Immunol.

[3] Remmers EF, Plenge RM, Lee AT, Graham RR, Hom G (2007). STAT4 and the risk of rheumatoid arthritis and systemic lupus erythematosus.. N Engl J Med.

[4] Thorburn CM, Prokunina-Olsson L, Sterba KA, Lum RF, Seldin MF (2007). Association of PDCD1 genetic variation with risk and clinical manifestations of systemic lupus erythematosus in a multiethnic cohort.. Genes Immun.

[5] Bauer JW, Baechler EC, Petri M, Batliwalla FM, Crawford D (2006). Elevated Serum Levels of Interferon-Regulated Chemokines Are Biomarkers for Active Human Systemic Lupus Erythematosus.. PLoS Med.

[6] Petri M (2000). Hopkins Lupus Cohort. 1999 update.. Rheum Dis Clin North Am.

[7] Criswell LA, Pfeiffer KA, Lum RF, Gonzales B, Novitzke J (2005). Analysis of families in the multiple autoimmune disease genetics consortium (MADGC) collection: the PTPN22 620W allele associates with multiple autoimmune phenotypes.. Am J Hum Genet.

[8] Demirci FYK, Manzi S, Ramsey-Goldman R, Minster RL, Kenney M (2006). Association of a common interferon regulatory factor 5 (IRF5) variant with increased risk of systemic lupus erythematosus (SLE).. Ann Hum Genet.

[9] Mitchell MK, Gregersen PK, Johnson S, Parsons R, Vlahov D (2004). The New York Cancer Project: rationale, organization, design, and baseline characteristics.. J Urban Health.

[10] Hom G, Graham RR, Modrek B, Taylor KE, Ortmann W (2008). Association of Systemic Lupus Erythematosus with C8orf13-BLK and ITGAM-ITGAX.. N Engl J Med.

[11] de Bakker PIW, Yelensky R, Pe'er I, Gabriel SB, Daly MJ (2005). Efficiency and power in genetic association studies.. Nat Genet.

[12] Barrett JC, Fry B, Maller J, Daly MJ (2005). Haploview: analysis and visualization of LD and haplotype maps.. Bioinformatics.

[13] Purcell S, Neale B, Todd-Brown KTL, Ferreira MAR, Bender D, Maller J, Sklar P, de Bakker PIW, Daly MJ, Sham PC (2007). PLINK: a toolset for whole-genome association and population-based linkage analysis.. American Journal of Human Genetics.

[14] Purcell S, Daly M, Sham P (2007). WHAP: haplotype-based association analysis.. Bioinformatics.

[15] Pritchard JK, Stephens M, Donnelly P (2000). Inference of population structure using multilocus genotype data.. Genetics.

[16] Seldin MF, Shigeta R, Villoslada P, Selmi C, Tuomilehto J (2006). European population substructure: clustering of northern and southern populations.. PLoS Genet.

[17] Price AL, Patterson NJ, Plenge RM, Weinblatt ME, Shadick NA (2006). Principal components analysis corrects for stratification in genome-wide association studies.. Nat Genet.

[18] R Development Core Team (2007). R: A language and environment for statistical computing. Vienna, Austria: R Foundation for Statistical Computing..

[19] Lee H-S, Remmers EF, Le J, Kastner DL, Bae S-C (2007). Association of STAT4 with Rheumatoid Arthritis in the Korean population.. Molecular Medicine.

[20] Jacob CO, Zang S, Li L, Ciobanu V, Quismorio F (2003). Pivotal role of Stat4 and Stat6 in the pathogenesis of the lupus-like disease in the New Zealand mixed 2328 mice.. J Immunol.

[21] Finnegan A, Grusby MJ, Kaplan CD, O'Neill SK, Eibel H (2002). IL-4 and IL-12 regulate proteoglycan-induced arthritis through Stat-dependent mechanisms.. J Immunol.

[22] Plenge RM, Padyukov L, Remmers EF, Purcell S, Lee AT (2005). Replication of putative candidate-gene associations with rheumatoid arthritis in >4,000 samples from North America and Sweden: association of susceptibility with PTPN22, CTLA4, and PADI4.. Am J Hum Genet.

[23] Lee AT, Li W, Liew A, Bombardier C, Weisman M (2005). The PTPN22 R620W polymorphism associates with RF positive rheumatoid arthritis in a dose-dependent manner but not with HLA-SE status.. Genes Immun.

[24] NCI-MHGRI Working Group on Replication in Association Studies (2007). Replicating genotype-phenotype associations.. Nature.

