# Supplementary figures and images for: Specificity of the STAT4 Genetic Association for Severe Disease Manifestations of Systemic Lupus Erythematosus
Source: PLoS Genet. 2008 May 30;4(5):e1000084. doi: 10.1371/journal.pgen.1000084 (PMC2377340; doi:10.1371/journal.pgen.1000084)

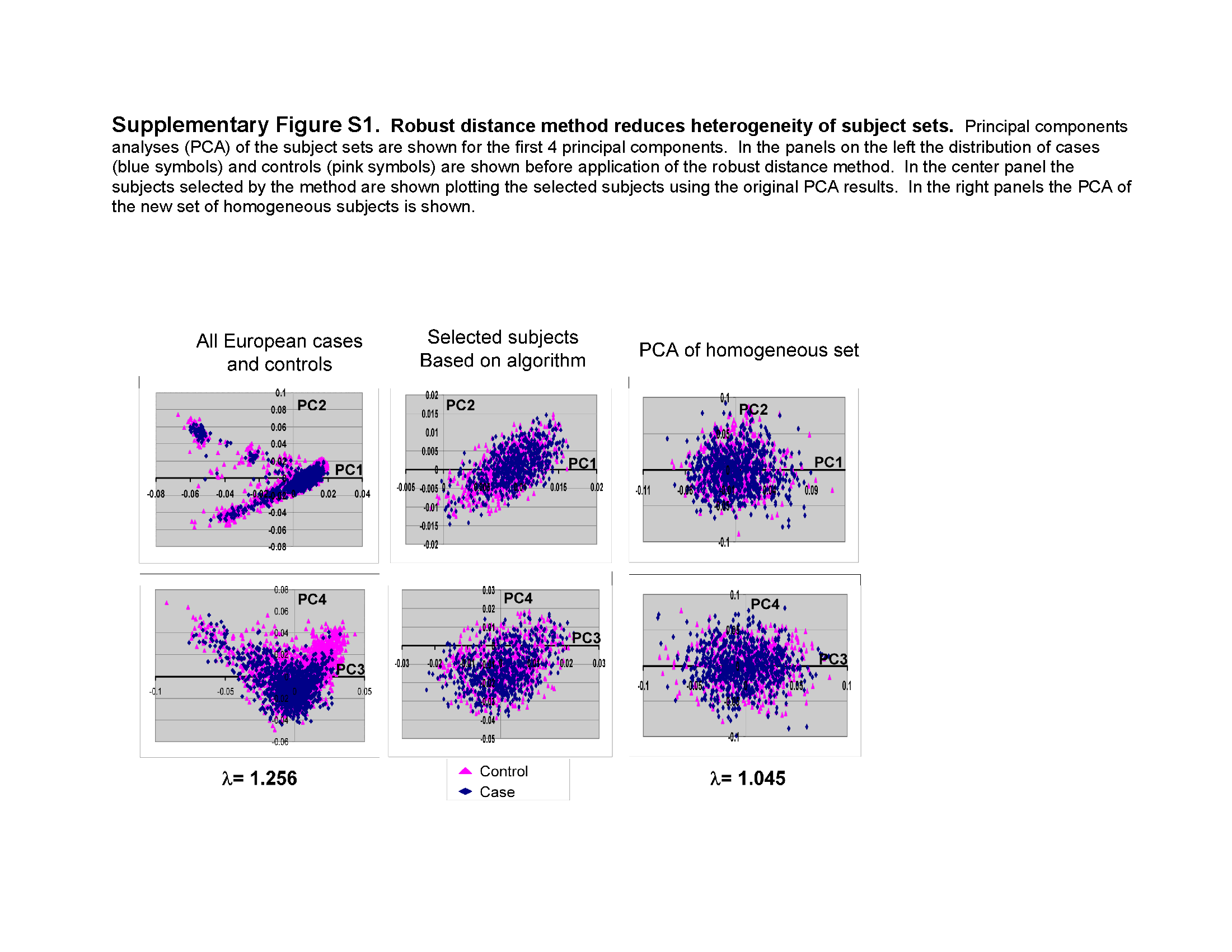

Supplement: Figure S1 — Robust distance method reduces heterogeneity of subject sets. (0.50 MB TIF) [file pgen.1000084.s001.tif]
