# Supplementary material for: Specificity of the STAT4 Genetic Association for Severe Disease Manifestations of Systemic Lupus Erythematosus
Source: PLoS Genet. 2008 May 30;4(5):e1000084. doi: 10.1371/journal.pgen.1000084 (PMC2377340; doi:10.1371/journal.pgen.1000084)
Supplement: Table S1 — MAFs and percent genotyping for all SNPs. (0.27 MB DOC) [file pgen.1000084.s002.doc]

| **Supplementary Table S1. Minor allele frequencies and percent genotyping for all SNPs on both Illumina 550K and Sequenom platforms.** Only SNPs passing quality-control criteria are included (MAF ≥ 5%, ≥ 90% genotyped, HWE p ≥ 0.001 in controls). | | | | | | |
| --- | --- | --- | --- | --- | --- | --- |
|  | | | | | | |
|  |  | **Illumina 550K** | | **Sequenom** | | |
| **SNP Name** | **Position** | **MAF** | **%Genotyped** | **MAF** | **% Genotyped** | |
| rs1860172 | 191526834 | 46.8% | 100.0% |  |  | |
| rs11685078 | 191529790 | 23.4% | 96.4% |  |  | |
| rs1882396 | 191541518 | 38.0% | 99.9% |  |  | |
| rs2355575 | 191553910 | 35.9% | 99.8% |  |  | |
| rs11685570 | 191554892 | 9.5% | 99.4% |  |  | |
| rs2204859 | 191562379 | 35.9% | 99.7% |  |  | |
| rs1921915 | 191577833 | 5.8% | 100.0% |  |  | |
| rs6758866 | 191581907 | 41.8% | 100.0% |  |  | |
| rs3771311 | 191596119 | 11.5% | 100.0% |  |  | |
| rs1921907 | 191610281 | 41.7% | 99.2% |  |  | |
| rs17748089 | 191615413 | 7.2% | 97.8% |  |  | |
| rs1517354 | 191641372 | 6.1% | 100.0% |  |  | |
| rs883844 | 191651275 | 37.8% | 99.9% |  |  | |
| rs3088307 | 191654918 |  |  | 43.6% | 99.9% | |
| rs12987796 | 191656373 | 14.8% | 99.5% |  |  | |
| rs3771300 | 191661102 | 47.7% | 100.0% |  |  | |
| rs16824035 | 191663140 |  |  | 15.8% | 99.9% | |
| rs6718902 | 191663710 | 26.4% | 99.5% | 24.8% | 99.8% | |
| rs13395505 | 191664020 | 41.6% | 100.0% | 40.7% | 99.2% | |
| rs17749316 | 191666215 |  |  | 7.3% | 99.6% | |
| rs13010343 | 191668951 | 12.3% | 100.0% |  |  | |
| rs1547550 | 191671231 |  |  | 34.8% | 99.2% | |
| rs2280234 | 191675605 |  |  | 40.8% | 99.9% | |
| rs2280233 | 191676072 |  |  | 44.7% | 99.8% | |
| rs2280232 | 191676272 | 25.0% | 99.7% | 26.6% | 100.0% | |
| rs13005843 | 191680232 |  |  | 7.9% | 99.7% | |
| rs11887698 | 191680380 | 17.3% | 99.9% | 18.2% | 100.0% | |
| rs7562024 | 191681027 | 43.8% | 99.8% |  |  | |
| rs12693590 | 191684142 | 7.2% | 98.7% |  |  | |
| rs12693591 | 191686008 | 14.7% | 99.9% |  |  | |
| rs16833157 | 191687904 | 6.6% | 100.0% |  |  | |
| rs13029247 | 191692164 | 32.5% | 99.9% |  |  | |
| rs10173099 | 191692838 | 35.9% | 99.8% |  |  | |
| rs10199181 | 191699059 |  |  | 41.2% | 99.7% | |
| rs13029532 | 191701407 | 7.5% | 99.9% | 8.2% | 100.0% | |
| rs10208033 | 191704923 |  |  | 45.8% | 94.9% | |
| rs1467199 | 191706008 |  |  | 24.7% | 96.6% | |
| rs6751855 | 191710277 | 49.5% | 98.4% |  |  | |
| rs16833177 | 191713138 |  |  | 20.6% | 99.6% | |
| rs6740131 | 191715088 | 21.3% | 100.0% |  |  | |
| rs4853456 | 191717269 |  |  | 20.1% | 100.0% | |
| rs3024912 | 191718593 | 25.2% | 99.8% |  |  | |
|  |  | **Illumina 550K** | | **Sequenom** | | |
| **SNP Name** | **Position** | **MAF** | **%Genotyped** | **MAF** | | **%Genotyped** |
| rs3024908 | 191719647 | 10.3% | 99.9% |  | |  |
| rs3024904 | 191720708 |  |  | 10.9% | | 99.9% |
| rs3024935 | 191721560 | 7.0% | 99.6% |  | |  |
| rs3024896 | 191722222 | 20.8% | 99.4% |  | |  |
| rs925847 | 191723046 | 28.8% | 99.8% | 28.0% | | 100.0% |
| rs6749371 | 191727690 |  |  | 6.6% | | 100.0% |
| rs3821236 | 191728264 | 22.3% | 98.8% |  | |  |
| rs16833215 | 191739305 | 32.7% | 100.0% | 32.5% | | 100.0% |
| rs16833220 | 191742850 |  |  | 15.9% | | 98.9% |
| rs3024866 | 191748347 |  |  | 27.6% | | 99.3% |
| rs932169 | 191754784 |  |  | 8.4% | | 99.5% |
| rs1517352 | 191756970 | 42.4% | 100.0% | 42.3% | | 99.9% |
| rs13017460 | 191758062 |  |  | 43.3% | | 99.7% |
| rs10168266 | 191761310 | 22.1% | 99.8% |  | |  |
| rs7594501 | 191764106 | 6.7% | 99.7% |  | |  |
| rs2459611 | 191764693 | 10.7% | 99.7% | 10.8% | | 99.6% |
| rs16833239 | 191765766 | 6.7% | 99.9% |  | |  |
| rs7601754 | 191765957 | 17.3% | 100.0% | 17.1% | | 98.6% |
| rs11889341 | 191769248 |  |  | 26.2% | | 100.0% |
| rs13010752 | 191772109 |  |  | 8.9% | | 99.6% |
| rs12998748 | 191774143 |  |  | 9.1% | | 97.5% |
| rs6434435 | 191779370 |  |  | 16.1% | | 100.0% |
| rs10931481 | 191780358 | 34.7% | 99.5% | 34.1% | | 99.7% |
| rs13011805 | 191781755 |  |  | 9.4% | | 100.0% |
| rs7574865 | 191790139 | 26.4% | 98.2% | 26.1% | | 99.5% |
| rs8179673 | 191794847 |  |  | 26.4% | | 99.9% |
| rs10181656 | 191795385 |  |  | 26.4% | | 100.0% |
| rs13401064 | 191795836 |  |  | 6.8% | | 99.9% |
| rs16833260 | 191797071 |  |  | 35.3% | | 99.7% |
| rs6752770 | 191799069 | 30.2% | 99.9% | 29.4% | | 98.7% |
| rs7599504 | 191801725 |  |  | 17.1% | | 99.7% |
| rs4341966 | 191807711 |  |  | 22.1% | | 99.6% |
| rs6738544 | 191814862 | 39.5% | 98.8% |  | |  |
| rs4555370 | 191816965 | 39.6% | 99.9% |  | |  |
| rs17769459 | 191819054 |  |  | 5.7% | | 100.0% |
| rs1551443 | 191822024 | 39.7% | 99.7% |  | |  |
| rs2356350 | 191828044 | 41.9% | 96.6% | 42.0% | | 99.5% |
| rs7596818 | 191834816 |  |  | 11.7% | | 99.9% |
| rs11685878 | 191834961 |  |  | 42.5% | | 100.0% |
| rs4853546 | 191835158 |  |  | 37.0% | | 99.9% |
| rs1031509 | 191835695 | 28.9% | 98.7% | 29.3% | | 99.4% |
| rs12327969 | 191836277 |  |  | 23.2% | | 99.9% |
| rs10497711 | 191839427 | 6.3% | 99.8% | 6.4% | | 99.8% |
| rs7572482 | 191840578 | 35.4% | 100.0% | 35.6% | | 99.4% |
| rs2278940 | 191841434 |  |  | 6.4% | | 100.0% |
|  |  | **Illumina 550K** | | **Sequenom** | | |
| **SNP Name** | **Position** | **MAF** | **%Genotyped** | **MAF** | | **%Genotyped** |
| rs897200 | 191843277 |  |  | 37.1% | | 99.7% |
| rs16833437 | 191844878 |  |  | 37.2% | | 100.0% |
| rs1031507 | 191846124 |  |  | 35.7% | | 99.9% |
| rs13001658 | 191850410 |  |  | 30.8% | | 99.8% |
| rs1869624 | 191855897 | 31.3% | 100.0% | 31.2% | | 91.6% |
| rs4853550 | 191856733 |  |  | 19.5% | | 99.8% |
| rs4853551 | 191856769 | 8.5% | 100.0% | 8.6% | | 99.1% |
| rs12467660 | 191857156 |  |  | 10.7% | | 99.9% |
| rs2054090 | 191859206 |  |  | 24.6% | | 99.6% |
| rs6434450 | 191864130 | 24.8% | 99.8% |  | |  |
| rs7595886 | 191864244 | 11.7% | 99.8% | 11.7% | | 99.9% |
| rs7606940 | 191870666 | 24.5% | 99.9% |  | |  |
| rs2356352 | 191877670 | 10.0% | 99.9% |  | |  |
| rs7560255 | 191884815 | 35.7% | 99.9% |  | |  |
| rs6709982 | 191886286 | 18.3% | 99.7% |  | |  |
| rs4853555 | 191897594 | 36.2% | 99.7% |  | |  |
| rs7556924 | 191906938 | 5.0% | 99.7% |  | |  |
| rs6434455 | 191910647 | 30.8% | 100.0% |  | |  |
| rs6728759 | 191912396 | 40.3% | 100.0% |  | |  |
| rs7574570 | 191924022 | 34.3% | 99.6% |  | |  |
| rs10445783 | 191927004 | 34.1% | 99.9% |  | |  |
| rs4853462 | 191949310 | 33.6% | 98.9% |  | |  |
| rs17439519 | 191955726 | 9.1% | 99.6% |  | |  |
| rs12989203 | 191966849 | 42.3% | 99.8% |  | |  |
| rs6732976 | 191971236 | 42.5% | 99.7% |  | |  |
| rs4853575 | 191986381 | 42.6% | 99.6% |  | |  |
| rs2067610 | 191989483 | 42.1% | 99.5% |  | |  |
| rs10497714 | 191992786 | 16.8% | 99.8% |  | |  |
| rs10931497 | 191996486 | 42.6% | 100.0% |  | |  |
| rs17348355 | 192001330 | 9.4% | 100.0% |  | |  |
| rs4613316 | 192016306 | 41.6% | 99.9% |  | |  |
| rs11892998 | 192030391 | 38.2% | 100.0% |  | |  |
| **Average** |  |  | **99.6%** |  | | **99.4%** |
