# Supplementary material for: Specificity of the STAT4 Genetic Association for Severe Disease Manifestations of Systemic Lupus Erythematosus
Source: PLoS Genet. 2008 May 30;4(5):e1000084. doi: 10.1371/journal.pgen.1000084 (PMC2377340; doi:10.1371/journal.pgen.1000084)
Supplement: Table S2 — SNP Concordance. (0.05 MB DOC) [file pgen.1000084.s003.doc]

**Supplementary Table S2. Concordance in subjects (n=1458) and SNPs with duplicate genotyping on Sequenom and Illumina 550K.**

| **SNP** | **%called 550K** | **%called Sequenom** | **%agreement** |
| --- | --- | --- | --- |
| rs6718902 | 99.53 | 96.37 | 99.86 |
| rs13395505 | 99.94 | 96.76 | 99.79 |
| rs2280232 | 99.63 | 96.68 | 100 |
| rs11887698 | 99.88 | 96.59 | 100 |
| rs13029532 | 99.81 | 97.75 | 99.86 |
| rs925847 | 99.81 | 97.71 | 99.79 |
| rs16833215 | 100 | 96.46 | 99.86 |
| rs1517352 | 100 | 96.59 | 99.86 |
| rs2459611 | 99.75 | 96.2 | 100 |
| rs7601754 | 99.94 | 95.25 | 100 |
| rs10931481 | 99.41 | 96.24 | 100 |
| **rs7574865** | **98.14** | **95.94** | **99.93** |
| rs6752770 | 99.88 | 95.21 | 99.71 |
| rs2356350 | 96.59 | 96.76 | 100 |
| rs7596818 | 99.78 | 97.71 | 100 |
| rs1031509 | 98.45 | 96.16 | 99.79 |
| rs10497711 | 99.84 | 97.54 | 99.93 |
| rs7572482 | 99.97 | 97.06 | 99.93 |
| rs1869624 | 99.97 | 89.21 | 100 |
| rs4853551 | 100 | 96.85 | 99.93 |
| rs7595886 | 99.78 | 96.5 | 99.65 |
| *Average* | *99.52857143* | *96.26380952* | *99.89952381* |
| *Minimum* | *96.59* | *89.21* | *99.65* |
