# Supplementary material for: Specificity of the STAT4 Genetic Association for Severe Disease Manifestations of Systemic Lupus Erythematosus
Source: PLoS Genet. 2008 May 30;4(5):e1000084. doi: 10.1371/journal.pgen.1000084 (PMC2377340; doi:10.1371/journal.pgen.1000084)
Supplement: Table S3 — SNP rs7574865 by ancestry. (0.03 MB DOC) [file pgen.1000084.s004.doc]

| **Supplementary Table S3. rs7574865 by ancestry, for 1731 study controls.** | | | |
| --- | --- | --- | --- |
|  |  |  |  |
| **Ancestry** | **N alleles** | **rs7574865 MAF** | **rs7574865 v. ancestry*** |
| European < 90% | 260 | 26.9% | p=0.11 |
| European ≥ 90% | 3202 | 22.4% |
| Northern European ≥ 90% | 1630 | 22.6% | p=0.77 |
| Northern European < 90% | 1570 | 22.1% |
| * Fisher's 2-sided exact test |  |  |  |
