# Supplementary material for: Specificity of the STAT4 Genetic Association for Severe Disease Manifestations of Systemic Lupus Erythematosus
Source: PLoS Genet. 2008 May 30;4(5):e1000084. doi: 10.1371/journal.pgen.1000084 (PMC2377340; doi:10.1371/journal.pgen.1000084)
Supplement: Table S4 — rs7574865 case-only associations. (0.04 MB DOC) [file pgen.1000084.s005.doc]

| | **Supplementary Table S4. rs7574865 association with phenotype status in Mantel-Haenszel combined cohorts.** | | | | | --- | --- | --- | --- | |  | | | | | **Phenotypes** | **p-value cohort heterogeneity** | **OR combined association** | **p-value combined association** | | **Severe nephritis**** | **0.996** | **1.50 (1.11 - 2.01)** | **0.0075** | | **Anti-dsDNA autoantibodies** | **0.81** | **1.44 (1.23 - 1.70)** | **1.0E-05** | | **First PC* > 0** | **0.28** | **1.43 (1.21 - 1.70)** | **3.0E-05** | | **Immunologic disorder** | **0.041** | **1.24 (1.04 - 1.49)** | **0.017** | | **Renal disorder** | **0.66** | **1.23 (1.03 - 1.46)** | **0.024** | | **Diagnosis < 30 years** | **0.55** | **1.22 (1.04 - 1.44)** | **0.018** | | Arthritis | 0.90 | 1.13 (0.92 - 1.38) | 0.25 | | Anti-nuclear autoantibodies | 0.38 | 1.11 (0.73 - 1.70) | 0.63 | | Hematologic disorder | 0.94 | 1.10 (0.93 - 1.30) | 0.28 | | Malar rash | 0.67 | 1.07 (0.91 - 1.26) | 0.39 | | Serositis | 0.53 | 1.03 (0.87 - 1.22) | 0.73 | | Photosensitivity | 0.18 | 1.00 (0.83 - 1.19) | 0.96 | | Second PC* > 0 | 0.38 | 0.98 (0.82 - 1.15) | 0.77 | | Neurologic disorder | 0.27 | 0.98 (0.74 - 1.29) | 0.86 | | Discoid rash | 0.79 | 0.95 (0.72 - 1.27) | 0.74 | | **Oral ulcers** | **0.74** | **0.80 (0.67 - 0.94)** | **0.0087** | | First p-value is Mantel-Haenzel test of homogeneity of association between the cohorts; followed by Mantel-Haenzel combined odds ratio (OR) and p-value. *Principal components of phenotypes (see Methods). **UCSF and ABCoN only. | | | | |
| --- | --- | --- | --- | --- | --- | --- | --- | --- | --- | --- | --- | --- | --- | --- | --- | --- | --- | --- | --- | --- | --- | --- | --- | --- | --- | --- | --- | --- | --- | --- | --- | --- | --- | --- | --- | --- | --- | --- | --- | --- | --- | --- | --- | --- | --- | --- | --- | --- | --- | --- | --- | --- | --- | --- | --- | --- | --- | --- | --- | --- | --- | --- | --- | --- | --- | --- | --- | --- | --- | --- | --- | --- | --- | --- | --- | --- | --- | --- | --- | --- |
